# Necking Reduction at Low Temperature in Aspect Ratio Etching of SiO_2_ at CF_4_/H_2_/Ar Plasma

**DOI:** 10.3390/nano14020209

**Published:** 2024-01-17

**Authors:** Hee-Tae Kwon, In-Young Bang, Jae-Hyeon Kim, Hyeon-Jo Kim, Seong-Yong Lim, Seo-Yeon Kim, Seong-Hee Cho, Ji-Hwan Kim, Woo-Jae Kim, Gi-Won Shin, Gi-Chung Kwon

**Affiliations:** Department of Electrical and Biological Physics, Kwangwoon University, 20 Kwangwoon-ro, Nowon-gu, Seoul 01897, Republic of Korea; heetae_kwon@outlook.com (H.-T.K.); bebe403@naver.com (I.-Y.B.); rlawogus6030@gmail.com (J.-H.K.); rlahyeonjo@naver.com (H.-J.K.); leemsy0628@naver.com (S.-Y.L.); rlatjdus323@naver.com (S.-Y.K.); sunghhee420@naver.com (S.-H.C.); oneaeo@hanmail.net (J.-H.K.); dnwo424@naver.com (W.-J.K.); swat2100@naver.com (G.-W.S.)

**Keywords:** low temperature, plasma etching, high aspect ratio, necking ratio, SiO_2_, trench, 3D NAND

## Abstract

This study investigated the effect of temperature on the aspect-ratio etching of SiO_2_ in CF_4_/H_2_/Ar plasma using patterned samples of a 200 nm trench in a low-temperature reactive-ion etching system. Lower temperatures resulted in higher etch rates and aspect ratios for SiO_2_. However, the plasma property was constant with the chuck temperature, indicated by the line intensity ratio from optical emission spectroscopy monitoring of the plasma. The variables obtained from the characterization of the etched profile for the 200 nm trench after etching were analyzed as a function of temperature. A reduction in the necking ratio affected the etch rate and aspect ratio of SiO_2_. The etching mechanism of the aspect ratio etching of SiO_2_ was discussed based on the results of the surface composition at necking via energy-dispersive X-ray spectroscopy with temperature. The results suggested that the neutral species reaching the etch front of SiO_2_ had a low sticking coefficient. The bowing ratio decreased with lowering temperature, indicating the presence of directional ions during etching. Therefore, a lower temperature for the aspect ratio etching of SiO_2_ could achieve a faster etch rate and a higher aspect ratio of SiO_2_ via the reduction of necking than higher temperatures.

## 1. Introduction

The current three-dimensional (3D) NAND flash technology is progressing toward expanding its architecture. This involves stacking an increasing number of deposited layers comprising alternating layers of stacked silicon (Si)-based materials, such as SiO_2_ and SiN, to lower the production costs per memory bit [1]. Considering the rapid development of device architectures, this technology is expected to ultimately enable the fabrication of stacks of 500–1000 layers [2]. When manufacturing such highly stacked layers, a high-aspect-ratio (HAR) etch is the most important process for achieving a device structure formed with trenches and holes having the desired aspect ratio (AR; depth/width > 100:1) in 3D NAND [2]. However, the current HAR etching methods, particularly when conducted at or near room temperature, present various challenges. One such challenge is low productivity resulting from the limited etching species at the etch front of the target materials with deeper features (i.e., a high aspect ratio (HAR)). The limited etching species reduce the etch rate of the target materials with the progress of HAR. Therefore, research and development efforts concerning the HAR process have attempted to overcome this problem using methods such as increasing the bias power, tuning the gas chemistry, and varying the substrate temperature [3,4,5]. Currently, the most promising method is the low-temperature HAR etching process [2,6]. This technology facilitates a high etch rate with higher AR structures compared with conventional HAR etching at room temperature [2]. However, the currently required low-temperature HAR etching process differs from the process typically used for Si because the low-temperature HAR etching process is aimed at Si-containing target materials such as SiO_2_ and SiN [2]. Recently, attempts have been made to study low-temperature etching. Recent studies focusing on etching thin films of SiO_2_ and SiN at low temperatures have shown intriguing results via different etching mechanisms compared to those at room temperature [7,8,9]. However, examples of related research on the aspect ratio etching of SiO_2_ are scarce. Although low-temperature etching of SiO_2_ as the target material in patterned samples with a large critical dimension (CD) (≥400 nm) has been conducted [10], the requirement of patterned samples with smaller CD in the low-temperature etching process must be investigated, considering the technology currently used in the semiconductor etch process. Furthermore, the low-temperature HAR etching process is expected to occur via a different mechanism. This is because plasma using leaner gas chemistry with higher fluorine and hydrogen concentrations, as well as the feature having HAR structure, is used for the low-temperature HAR etching of Si-containing target materials, such as SiO_2_ and SiN [2].

Therefore, this study investigated the effect of temperature on the aspect ratio etching of SiO_2_ in CF_4_/H_2_/Ar plasma using patterned samples of a 200 nm trench in a low-temperature reactive ion etching system. The lower temperature resulted in a higher aspect ratio and etch rate of SiO_2_. The results for the variables obtained from the characterization of the etched profile for the 200 nm trench were analyzed as a function of temperature. A reduction in the necking ratio was observed with lowering temperature. Furthermore, the etching mechanism of the aspect ratio etching of SiO_2_ was discussed, along with the results of the surface composition at necking via energy-dispersive X-ray spectroscopy (EDS) with temperature. The bowing and necking ratios exhibited a clear dependence on temperature. Further, the aspect ratio contact etching of SiO_2_ was conducted for a 200 nm trench.

## 2. Materials and Methods

**Figure 1:**
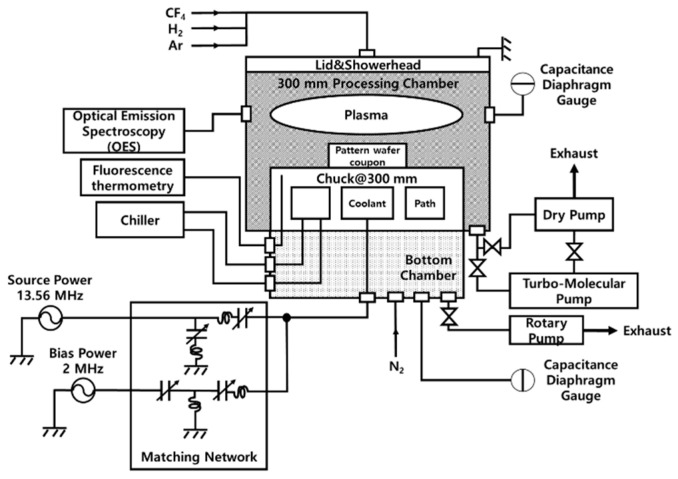
Schematic of the 300 mm low-temperature reactive ion etch system used for the experiments.

The experimental setup of the 300 mm low-temperature reactive ion etching system is shown in Figure 1. The system comprised a 300 mm processing chamber and a bottom chamber. The purpose of the bottom chamber was to prevent condensation on the bottom surface of the chuck. The vacuum system of the bottom chamber was independent of that of the 300 mm processing chamber. The 300 mm chuck was accommodated in a 300 mm processing chamber. The temperature of the chuck was measured using a fluorescence thermometer installed in the bottom chamber with feedthrough. Here, 13.56 MHz (continuous wave, CW) and 2 MHz (CW) radio frequency (RF) generators were used to supply RF power to the chuck through a matching network (Path Finder, PLASMART, Seoul, Republic of Korea) via the bottom chamber. The gap between the chuck and a showerhead was 50 mm. The chuck temperature was reduced using a chiller. The coolant supply and return lines from the chuck were installed such that they passed through the bottom chamber. Optical emission spectroscopy (OES) was used to monitor the plasma properties (HR4000 spectrometer, Ocean Optics, Dunedin, FL, USA) through the wall of a 300 mm processing chamber. The OES was positioned 18 mm from the chuck along the gap axis. The gas mixture comprising CF_4_/H_2_/Ar, wherein the flow was controlled by a mass flow controller (MFC, MARU series, MKP, Yongin-si, Republic of Korea) and an integrated gas system (IGS, UNILOK, Changwon, Republic of Korea), was injected into the 300 mm processing chamber through a lid equipped with the showerhead. During the injection of the gas mixture into the 300 mm processing chamber, the pressure of this processing chamber was controlled and maintained using a pendulum valve and turbo-molecular pump.

The experimental conditions were as follows. The total flow rate of the gas mixture was 400 sccm; the flow rate of Ar was fixed at 200 sccm, and that of (CF_4_+H_2_) was fixed at 200 sccm. The proportion of hydrogen [H_2_/(CF_4_+H_2_)] was fixed at 35%. The pressure of the 300 mm processing chamber was 25 mTorr. The RF powers at the frequencies of 13.56 and 2 MHz were 600 and 700 W, respectively. Under these conditions, V_dc_ (monitored by the matching network) remained at −1000 V. The pressure of the bottom chamber was maintained at 550 Torr to prevent the arcing of the high RF power with N_2_ gas and a rotary vane pump. The etching time was set to 6 min. O_2_ plasma was used to clean the 300 mm processing chamber after each etching process. The abovementioned experimental conditions were used to etch patterned samples of a 200 nm trench with CF_4_/H_2_/Ar plasma.

**Figure 2:**
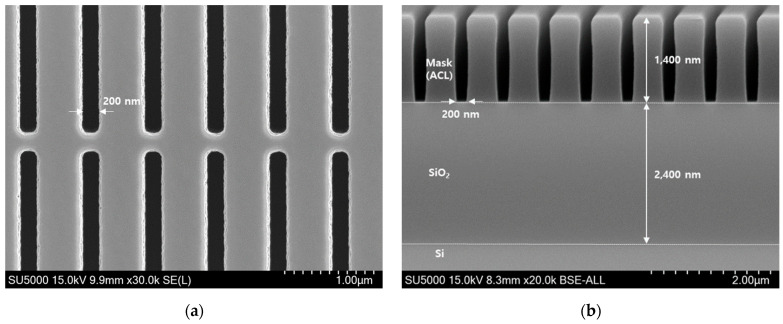
(**a**) SEM image of 200 nm trench before etching for top view; (**b**) SEM image of 200 nm trench before etching for cross-sectional view.

Figure 2 shows the top and cross-sectional images of the patterned sample for the 200 nm trench before etching. SiO_2_ with a thickness of 2400 nm was deposited on the Si substrate. An amorphous carbon layer (ACL) mask with a thickness of 1400 nm was deposited on SiO_2_. The patterned sample (26 × 28 mm) was placed on the chuck surface, which was in close proximity to the position at which the chuck temperature was measured using fluorescence thermometry with vacuum grease for thermal conduction. The effect of bowing at the mask, shown in Figure 2 on the results obtained after etching was not considered because the samples used in the experiments were the same. The patterned samples used in the etching experiments were supported by the Korea Semiconductor Industry Association (KSIA, Seongnam, Republic of Korea).

After etching the patterned samples, scanning electron microscopy (SEM, Hitachi SU-5000 FE-SEM, Tokyo, Japan) was used to characterize the etched profile of the samples for the 200 nm trench. The surface composition at necking was analyzed using EDS (Oxford Instruments, Abingdon, UK).

**Figure 3:**
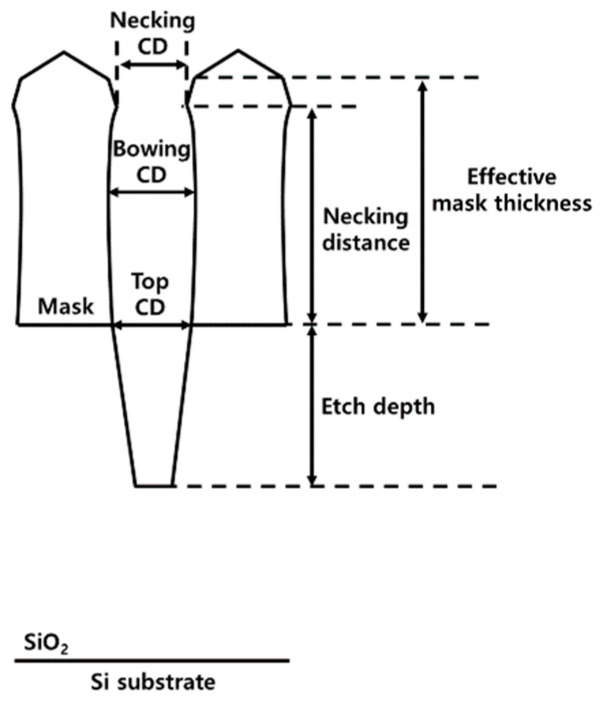
Characterization of the etched profile for 200 nm trench after etching to obtain variables.

In Figure 3, the top CD is the width at the boundary between the SiO_2_ and the mask (i.e., the ACL) after etching; the bowing CD is the maximum width after etching; the necking CD is the minimum width after etching; the necking distance is the distance from the boundary to the location of necking CD after etching; the etch depth is the distance from the boundary to the total depth of SiO_2_ after etching; and the effective mask thickness after etching is the mask thickness without a facet. The variables in Equations (1)–(6) were obtained to determine the effect of temperature on the aspect ratio etching of SiO_2_:

The etch rate of SiO_2_ is as follows:

(1) $$\frac{Etch\;depth}{Etch\;time}$$

The etch rate of mask is as follows:

(2) $$\frac{\left(1400\;nm-effective\;mask\;thickness\right)}{Etch\;time}$$

The aspect ratio of SiO_2_ is as follows:

(3) $$\frac{Etch\;depth}{Top\;CD}$$

Necking distance over effective mask thickness is expressed as follows:

(4) $$\frac{Necking\;distance}{Effective\;mask\;thickness}$$

Necking ratio is as follows:

(5) $$\frac{\left(Top\;CD-Necking\;CD\right)}{Top\;CD}$$

Bowing ratio is as follows:

(6) $$\frac{\left(Bowing\;CD-Top\;CD\right)}{Top\;CD}$$

## 3. Results and Discussion

**Figure 4:**
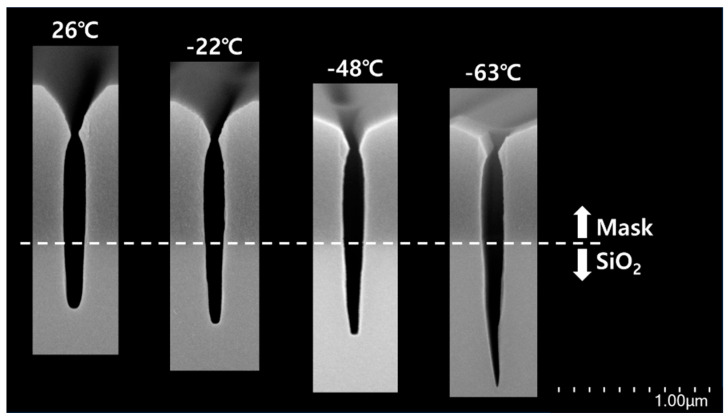
SEM images of the etched profiles of 200 nm trench with chuck temperature from 26 °C to −63 °C.

**Figure 5:**
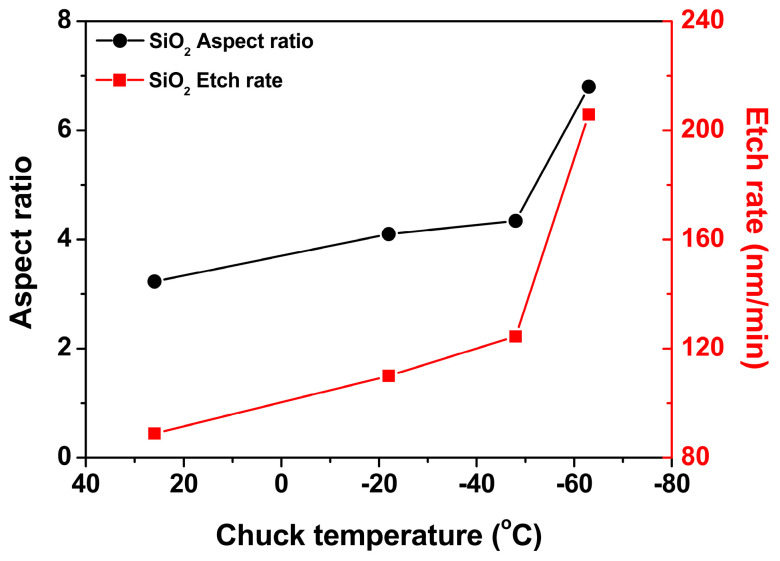
Aspect ratio and etch rate of SiO_2_ as a function of chuck temperature from the SEM images in Figure 4.

**Figure 6:**
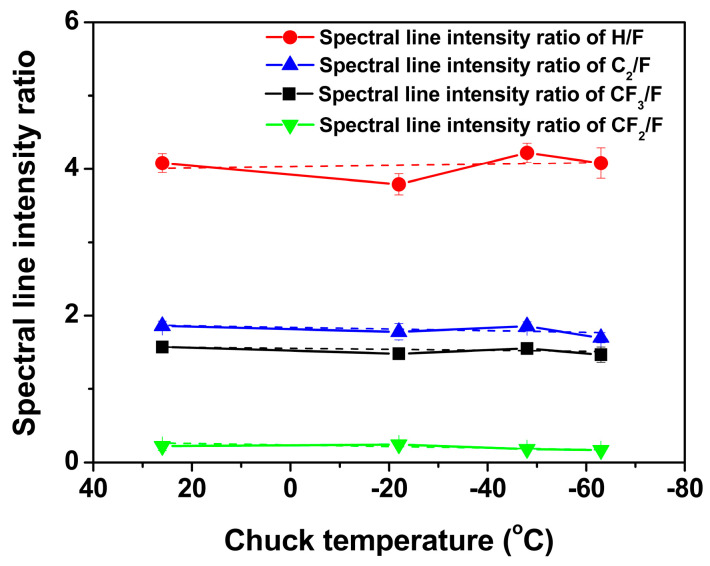
Spectral line intensity ratio of H/F, C_2_/F, CF_3_/F, and CF_2_/F as a function of chuck temperature.

Based on the SEM results shown in Figure 4, the aspect ratio and etch rate of SiO_2_ were analyzed as a function of the chuck temperature, as shown in Figure 5. The chuck temperature is the temperature at the steady state. Figure 5 shows that both the aspect ratio and etch rate of SiO_2_ increased as the temperature decreased. The effect of the change of the plasma property with the temperature on the aspect ratio and the etch rate of SiO_2_ was investigated. Thus, the spectral line intensity ratio in Figure 6 was obtained by monitoring the spectral line intensity of species (CF_3_ (610.8 nm), CF_2_ (340 nm), F (703.8 nm), C_2_ (516.5 nm), and H (486.1 nm)) from CF_4_/H_2_/Ar plasma during the aspect ratio etching of SiO_2_ using OES [11,12,13]. As the spectral line intensity ratio CF/F was excessively low, it is not shown in Figure 6. However, CF was formed in plasma by a CF_4_/H_2_ plasma chemical reaction [14,15]. The spectral line intensity ratio of C_2_/F in Figure 6 indicates that the plasma properties were depositive [13]. From the data shown in Figure 6, the plasma properties were believed to remain constant as a function of temperature.

**Figure 7:**
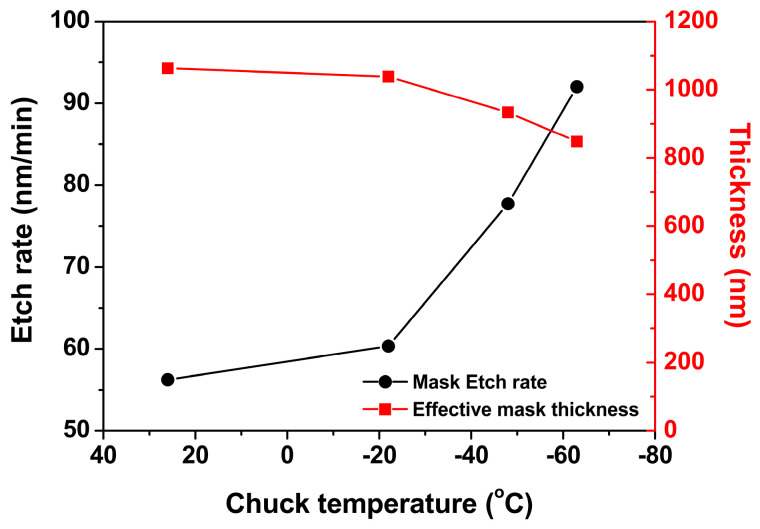
Mask etch rate and effective mask thickness as a function of chuck temperature from the SEM images in Figure 4.

Because the plasma properties were assumed to be independent of the chuck temperature, analysis of the etched profiles was further performed as a function of the chuck temperature. Figure 7 shows that effective mask thickness decreased from the temperature of 26 °C to −63 °C. Regarding the mask thickness, from the spectral line intensity ratio of C_2_/F shown in Figure 6, a polymer appeared to be deposited on the mask. However, as indicated by the decrease in the effective mask thickness from the temperature of 26 °C to −63 °C, the etch rate of the mask increased (Figure 7). The increase in the etch rate of the mask could be ascribed to the lower temperature, which induced an increase in the density of fluorine near the feature. The density of fluorine near the wafer increased at lower wafer temperatures [16]. The increase in the density of fluorine, which can act as a radical for the etching of the mask near the feature, increased the etch rate of the mask [17].

**Figure 8:**
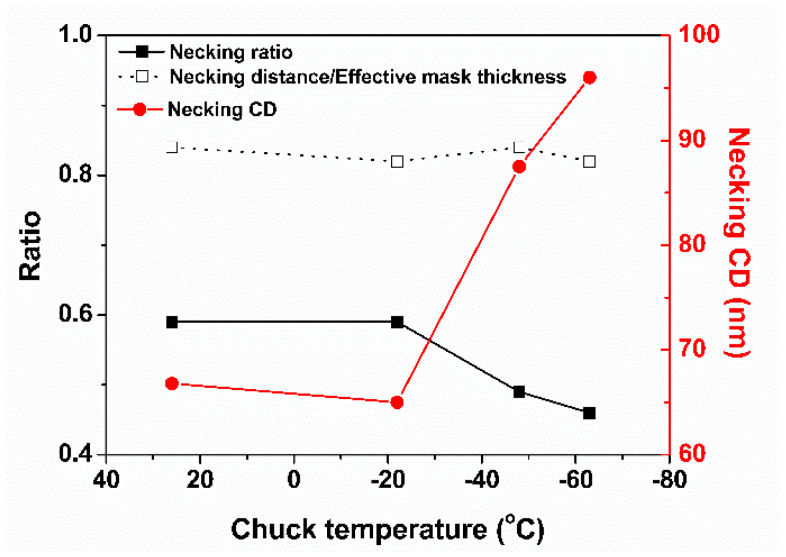
Necking ratio, necking distance/effective mask thickness, and necking critical dimension (CD) as a function of chuck temperature from the SEM images in Figure 4.

Each necking distance was divided by each effective mask thickness with chuck temperature from 26 °C to −63 °C, respectively, as shown in Figure 8. Figure 8 shows that the necking distance over the effective mask thickness appeared to be constant as a function of temperature. Both the necking CD and necking ratio are shown as a function of temperature in Figure 8. The necking CD tended to widen at lower temperatures. However, the necking ratio decreased. The factor influencing the increase in both the aspect ratio and etch rate of SiO_2_ in Figure 5 is the reduction of necking at a lower temperature, which enhanced the fluxes of neutrals (including radicals) and ions onto the etch front of SiO_2_ [18]. Specifically, it is believed that the flux of neutrals onto the etch front of SiO_2_ was more enhanced at lower temperatures than the flux of ions [6]. The flux of ions was independent of the CD of the pattern and temperature rather than that of neutrals [16,19]. Therefore, the etched profile of SiO_2_ can also be described. The etched profile of SiO_2_ shown in Figure 4 became tapered as the chuck temperature decreased. This tapered profile was attributed to an increase in the flux of neutrals at lower temperatures [20]. Accordingly, it is reasonable that the etched profile of SiO_2_ exhibited a tapered profile at the lower temperature, shown in Figure 4, in that the aspect ratio etching with a lower temperature was fundamentally aimed at enhancing the neutral fluxes onto the etch front at the bottom of the high-aspect-ratio structure.

**Figure 9:**
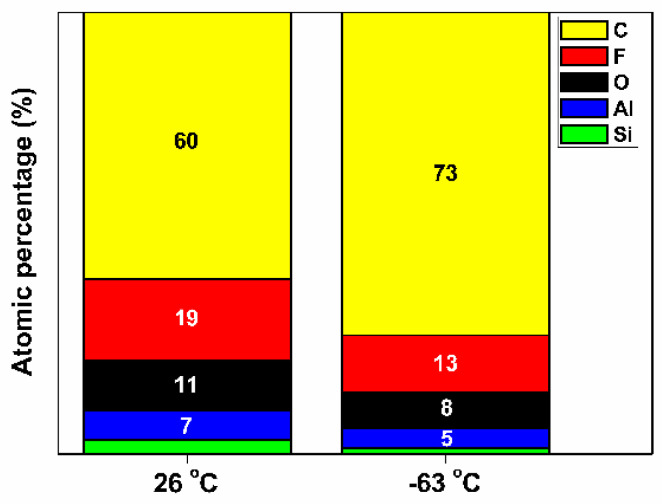
Atomic percentage of the surface at necking from energy-dispersive X-ray spectroscopy (EDS) with chuck temperature of 26 °C and −63 °C, respectively. (The atomic percentage of Si, which is not shown in Figure 9 for the temperature of both 26 °C and −63 °C, is specified in the content).

To determine the neutral species with greater participation in the aspect ratio etching of SiO_2_, the surface composition at necking was analyzed via EDS with chuck temperatures of 26 °C and −63 °C. Figure 9 shows the atomic percentage of the surface at necking from EDS with respect to temperature. Considering the formation of (hydro)fluorocarbons in the plasma, the major composition of the surface at necking can be considered as mainly carbon (C) and fluorine (F). The appearance of oxygen (O), together with silicon (Si) and aluminum (Al), originated from the environment before the measurement and chamber components, respectively [21,22]. The atomic percentages of Si at chuck temperatures of 26 °C and −63 °C were 3% and 1%, respectively. Nevertheless, the atomic percentages of O, Al, and Si at each temperature were significantly lower than the combined atomic percentages of carbon and fluorine in Figure 9. Therefore, the most noticeable observation was that the atomic percentage of C increased with increasing temperature. However, the atomic percentage of F decreased with temperature. The clear difference in the atomic percentages of C and F with temperature was owing to the sticking coefficient of the (hydro)fluorocarbons. The sticking coefficient of a species increases as the temperature lowers [5,23]. The reaction probabilities of fluorine and hydrogen were not considered to be dependent on the temperature under the experimental conditions [16,23]. Consequently, (hydro)fluorocarbon species with a relatively high sticking coefficient reached the etch front of SiO_2_ with a low probability. In other words, the species with a relatively low sticking coefficient reached the etch front of SiO_2_ with a high probability. Consequently, the neutral species participating in the etching of SiO_2_ at the etch front in the aspect ratio etching of SiO_2_ were considered to be CF_3_, H, and F, with a high probability from the results of the spectral line intensity ratio in Figure 6. The etching mechanism of the SiO_2_ film in CF_4_/H_2_ plasma also reached a similar conclusion [8].

**Figure 10:**
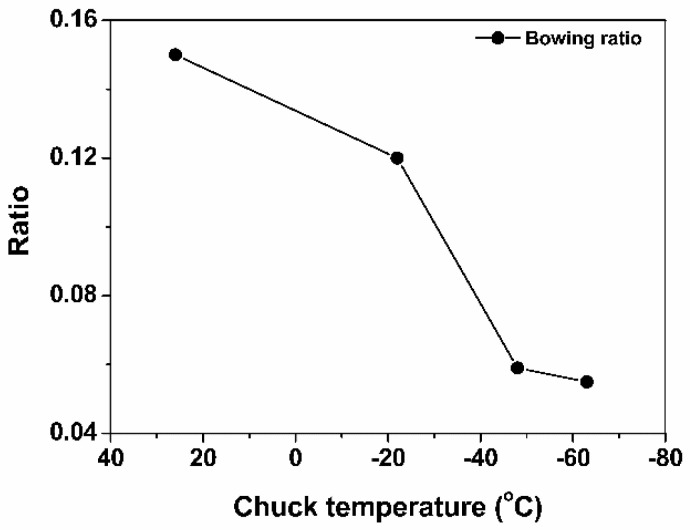
Bowing ratio as a function of chuck temperature from the SEM images in Figure 4.

As shown in Figure 8, the necking ratio decreased with lowering temperature, and the bowing ratio was confirmed. Figure 10 shows the decrease in the bowing ratio with temperature. Therefore, the necking ratio in Figure 8 is reasonable because bowing became severe as necking evolved [24]. Therefore, it is expected that the ions will become more directional with decreasing temperature because the bowing ratio indicates the degree of ion scattering inside the feature during etching [23,25]. Consequently, the increase in both the etch rate and aspect ratio of SiO_2_ with decreasing temperature in Figure 5 is attributed to the reduction of necking at lower temperatures.

**Figure 11:**
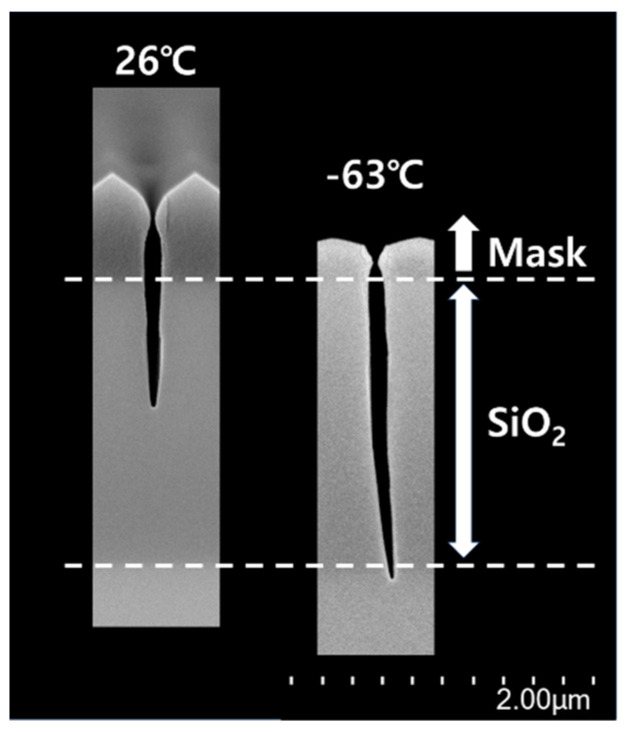
SEM images of the etched profiles for 200 nm trench at chuck temperature of 26 °C and −63 °C, respectively, at the same experimental condition as the previous results, only adopting a longer process time.

Further experiments at the temperatures of 26 °C and −63 °C were conducted under the same experimental conditions as those for 6 min but with the process time changed to 15 min. Figure 11 shows the aspect ratio contact etching of SiO_2_ at a 200 nm trench with a temperature of −63 °C. Both the etching rate of SiO_2_ and the selectivity for SiO_2_ over the mask, presented in Table 1, were obtained from Figure 11. Table 1 shows the increase in both the etch rate of SiO_2_ and the selectivity when the temperature was changed from 26 °C to −63 °C. Therefore, a lower temperature for the aspect-ratio contact etching of SiO_2_ achieved a higher etch rate, leading to improved productivity. However, the etched profile at the temperature of −63 °C presented a twisting. Research on twisting has not been conducted yet. Attempts to improve the etched profile will be conducted in future studies.

## 4. Conclusions

The effect of temperature on the aspect-ratio etching of SiO_2_ in CF_4_/H_2_/Ar plasma was investigated using patterned samples of a 200 nm trench in a low-temperature reactive-ion etching system. Both the aspect ratio and etch rate of SiO_2_ were increased by lowering the chuck temperature. The spectral line intensity ratio results confirmed that the plasma properties did not change with temperature. Therefore, the factors influencing the etch rate and aspect ratio of SiO_2_ were determined by characterizing the etched profile of the 200 nm trench. The reduction of necking by lowering the temperature increased the aspect ratio and etch rate of SiO_2_. The surface composition at necking was analyzed via EDS for temperatures of 26 °C and −63 °C. The EDS results were discussed with respect to the etching mechanism of the aspect ratio etching of SiO_2_. The neutrals reaching the etch front of SiO_2_ were considered to be species with relatively low sticking coefficients, such as CF_3_, H, and F, at lower temperatures. The bowing ratio, as well as the necking ratio, decreased by lowering the temperature. Therefore, a lower temperature facilitated both a higher aspect ratio and higher etch rate of SiO_2_ by reducing necking. The aspect ratio contact etching of SiO_2_ was further conducted for a 200 nm trench. Lower temperatures resulted in a faster etching rate than higher temperatures, although twisting at the bottom of the etched profile occurred at lower temperatures. The etched profiles will be further improved in future studies.

## Figures and Tables

**Table 1:** Comparison between the chuck temperatures of 26 °C and −63 °C for the etch rate of SiO_2_ and selectivity from the SEM images in Figure 11.

	Chuck Temperature (°C)	
	26	−63
Etch rate of SiO_2_ (nm/min)	69	158
Selectivity	1.5	2.2

## Data Availability

Data available on reasonable request.

## References

[1] Park K.T., Byeon D.S., Kim D.H. A world’s first product of three-dimensional vertical NAND Flash memory and beyond. Proceedings of the 2014 14th Annual Non-Volatile Memory Technology Symposium (NVMTS).

[2] Shen M., Lill T., Hoang J., Chi H., Routzahn A., Church J., Subramonium P., Puthenkovilakam R., Reddy S., Bhadauriya S. (2023). Progress report on high aspect ratio patterning for memory devices. Jpn. J. Appl. Phys..

[3] Ishikawa K., Karahashi K., Ishijima T., Cho S.I., Elliott S., Hausmann D., Mocuta D., Wilson A., Kinoshita K. (2018). Progress in nanoscale dry processes for fabrication of high-aspect-ratio features: How can we control critical dimension uniformity at the bottom?. Jpn. J. Appl. Phys..

[4] Iwase T., Yokogawa K., Mori M. (2018). Eliminating dependence of hole depth on aspect ratio by forming ammonium bromide during plasma etching of deep holes in silicon nitride and silicon dioxide. Jpn. J. Appl. Phys..

[5] Tandou T., Kubo S., Yokogawa K., Negishi N., Izawa M. (2016). Improving the etching performance of high-aspect-ratio contacts by wafer temperature control. Precis. Eng..

[6] Lill T., Berry I.L., Shen M., Hoang J., Fischer A., Panagopoulos T., Chang J.P., Vahedi V. (2023). Dry etching in the presence of physisorption of neutrals at lower temperatures. J. Vac. Sci. Technol. A.

[7] Dussart R., Ettouri R., Nos J., Antoun G., Tillocher T., Lefaucheux P. (2023). Cryogenic etching of silicon compounds using a CHF_3_ based plasma. J. Appl. Phys..

[8] Hsiao S.-N., Britun N., Nguyen T.T.N., Tsutsumi T., Ishikawa K., Sekine M., Hori M. (2023). Manipulation of etch selectivity of silicon nitride over silicon dioxide to a-carbon by controlling substate temperature with a CF_4_/H_2_ plasma. Vacuum.

[9] Hattori T., Kobayashi H., Ohtake H., Akinaga K., Kurosaki Y., Takei A., Sekiguchi A., Maeda K., Takubo C., Yamada M. (2023). Highly selective isotropic gas-phase etching of SiO_2_ using HF and methanol at temperatures −30 °C and lower. Jpn. J. Appl. Phys..

[10] Sato M., Takehara D., Uda K., Hara K.S. (1992). Suppression of microloading effect by low-temperature SiO_2_ etching. Jpn. J. Appl. Phys..

[11] Kim J., Choi G., Kwon K.H. (2023). High-aspect-ratio oxide etching using CF_4_/C_6_F_12_O plasma in an inductively coupled plasma etching system with low-frequency bias power. Plasma Process. Polym..

[12] Jin D.Z., Yang Z.H., Tang P.Y., Xiao K.X., Dai J.Y. (2008). Hydrogen plasma diagnosis in penning ion source by optical emission spectroscopy. Vacuum.

[13] Fukasawa T., Nakamura A., Shindo H., Yasuhiro Horiike Y.H. (1994). High rate and highly selective SiO_2_ etching employing inductively coupled plasma. Jpn. J. Appl. Phys..

[14] Montazer Rahmati P.M., Arefi F., Amouroux J. (1991). Plasma polymerization of CF_4_+ H_2_ mixtures on the surface of polyethylene and polyvinylidene flouride substrates. Surf. Coat. Technol..

[15] Ryan K.R., Plumb I.C. (1984). Gas-phase reactions of CF_3_ and CF_2_ with hydrogen atoms: Their significance in plasma processing. Plasma Chem. Plasma Process..

[16] Tinck S., Tillocher T., Dussart R., Bogaerts A. (2015). Cryogenic etching of silicon with SF_6_ inductively coupled plasmas: A combined modelling and experimental study. J. Phys. D Appl. Phys..

[17] Li J., Kim Y., Han S., Chae H. (2021). Ion-enhanced etching characteristics of sp^2^-rich hydrogenated amorphous carbons in CF_4_ plasmas and O_2_ plasmas. Materials.

[18] Coburn J.W., Winters H.F. (1989). Conductance considerations in the reactive ion etching of high aspect ratio features. Appl. Phys. Lett..

[19] Doemling M.F., Rueger N.R., Oehrlein G.S. (1996). Observation of inverse reactive ion etching lag for silicon dioxide etching in inductively coupled plasmas. Appl. Phys. Lett..

[20] Huard C.M., Zhang Y., Sriraman S., Paterson A., Kushner M.J. (2017). Role of neutral transport in aspect ratio dependent plasma etching of three-dimensional features. J. Vac. Sci. Technol. A.

[21] Cheng Q.J., Long J.D., Chen Z., Xu S. (2007). Chemically active plasmas for deterministic assembly of nanocrystalline SiC film. J. Phys. D Appl. Phys..

[22] Zeze D.A., Carey J.D., Stolojan V., Weiss B.L., Silva S.R.P. (2006). Damage effects in Pyrex by CF_4_ reactive ion etching in dual RF-microwave plasmas. Micro Nano Lett..

[23] Izawa M., Negishi N., Yokogawa K.E., Momonoi Y. (2007). Investigation of bowing reduction in SiO_2_ etching taking into account radical sticking in a hole. Jpn. J. Appl. Phys..

[24] Kim D., Hudson E.A., Cooperberg D., Edelberg E., Srinivasan M. (2007). Profile simulation of high aspect ratio contact etch. Thin Solid Film..

[25] Rangelow I.W. (2003). Critical tasks in high aspect ratio silicon dry etching for microelectromechanical systems. J. Vac. Sci. Technol. A.

